# Long- term outcome of paediatric patients with ANCA vasculitis

**DOI:** 10.1186/1546-0096-9-12

**Published:** 2011-06-19

**Authors:** Nishkantha Arulkumaran, Susan Jawad, Stuart W Smith, Lorraine Harper, Paul Brogan, Charles D Pusey, Alan D Salama

**Affiliations:** 1 Renal Section, Division of Medicine, Imperial College London, Hammersmith Hospital, Ducane Road, London W12 0NN, UK; 2 Division of Renal Immunobiology, Medical School, West Extension, 1st Floor, University of Birmingham, Edgbaston, Birmingham, B15 2TT, UK; 3 Department of Rheumatology, Institute of Child Health, Great Ormond Street Hospital, 30 Guilford St, London WC1N 1EH, UK; 4 Centre for Nephrology, University College London, Royal Free Hospital, London NW3 2PF, UK

## Abstract

**Background:**

Primary systemic vasculitis presenting in childhood is an uncommon but serious condition. As these patients transfer to adult clinics for continuing care, defining long term outcomes with emphasis on disease and treatment- related morbidity and mortality is important. The aim of this study is to describe the long- term clinical course of paediatric patients with ANCA vasculitis.

**Methods:**

The adult patients in our vasculitis clinics who had presented in childhood, with a follow up time of greater than 10 years were included. We also reviewed the literature for articles describing the clinical outcome of paediatric patients with ANCA vasculitis.

**Results:**

We describe the clinical course of 8 adults who presented in childhood with ANCA vasculitis. 7 patients had Wegener's granulomatosis and 1 had microscopic polyangiitis. The median age at presentation was 11.5 years, and follow up time ranged form 11 to 30 years. Induction therapy for all patients was steroids and/or cyclophosphamide. Maintenance therapy was with azathioprine or mycophenolate mofetil. Biological agents were used in 3 patients for relapsed disease in adulthood only.

Seven patients achieved complete remission. All patients experienced disease relapse, with a median of 4 episodes. Kidney function was generally well preserved, with median eGFR 76 ml/min. Only one patient developed end-stage renal failure and one patient died after 25 years of disease. Treatment-related morbidity rates were high; 7 suffered from infections, 4 were infertile, 2 had skeletal complications, and 1 developed malignancy.

**Conclusion:**

Close long- term follow up of paediatric patients with ANCA vasculitis is imperative, as this patient cohort is likely to live long enough to develop significant treatment and disease- related morbidities. Prospective cohort studies with novel therapies including paediatric patients are crucial to help us determine the best approach to managing this complex group of patients. In addition, although not yet observed in our series, late cardiovascular morbidity remains a major longer-term potential concern for adult survivors of paediatric vasculitis.

## Background

Primary systemic vasculitides, with the exception of Henoch Schönlein purpura (HSP) and Kawasaki disease (KD), are diseases that predominantly affect people later in adulthood, typically in their sixth and seventh decades [1]. Vasculitides such as Wegener's granulomatosis (WG) and polyarteritis nodosa (PAN) have different aetiological, clinical, and prognostic features in children [2,3]. While there are numerous studies describing the long- term outcome in adults with ANCA vasculitis, to date the vast majority of published studies on paediatric patients with ANCA vasculitis have a limited follow up, at which stage they may be transferred to the adult vasculitis service. Furthermore, there are few data on the progression of paediatric ANCA vasculitis patients into adulthood, which is primarily due to the rare occurrence of these disease entities in childhood.

The commonest childhood vasculitides are HSP and KD, the estimated incidence of which is 20 per 100 000 children and 8 per 100, 000 children under 5 years in the UK population respectively [4,5]. By contrast the incidence of paediatric WG and microscopic polyangiitis (MPA) is significantly less or unknown. The estimated incidence of childhood WG is less than 1 per 2 million per year [6], and MPA incidence has not been determined in paediatric patients.

In this retrospective study, we reviewed the clinical features of 8 children diagnosed with ANCA vasculitis and the progression of their disease into adulthood. Diagnoses were made based on clinical criteria, laboratory testing, and histology, as well as the EULAR/PReS proposed consensus criteria for the classification of childhood vasculitides [7].

The objective of the study was to determine the progression of the clinical features of childhood ANCA vasculitis into adulthood, and to determine the long- term disease and treatment- related morbidities. Particular emphasis was placed on establishing long- term complications, and the total burden of immunosuppression, in relation to relapse rate and treatment- related morbidity.

In addition, a literature review was performed to better determine the common clinical features and outcome of these patients.

## Methods

We performed a retrospective study of patients attending the adult vasculitis clinic at Hammersmith Hospital, London and the University Hospital of Birmingham. From the vasculitis databases, patients with ANCA vasculitis who presented before the age of 16 years were identified. Only those who had been followed for more than 10 years were included in our cohort.

Patient demographics and clinical course (age of presentation, clinical features on initial and subsequent presentations, immunosuppression, complications of treatment and the underlying disease and relapse rate) were determined from the case notes. Results of biochemical, histological, and radiological investigations were determined from the hospital patient records database.

ANCA associated vasculitis was diagnosed according to the EULAR/PReS proposed consensus criteria for the classification of childhood vasculitides [7,8], summarised below. In brief, a diagnosis of Wegener's granulomatosis (WG, now termed granulomatosis with polyangiitis) required three of the following features; abnormal urinalysis, granulomatous inflammation on biopsy, nasal sinus inflammation, subglottic, tracheal, or endobronchial stenosis, an abnormal chest *X-*ray or CT and PR3-ANCA or C-ANCA staining by indirect immunofluorescence. A diagnosis of microscopic polyangiitis (MPA) was based on a clinical constellation of necrotising pauci-immune vasculitis affecting predominantly small vessels associated with a high titre of MPO-ANCA or positive p-ANCA staining, necrotising glomerulonephritis, or pulmonary capillaritis in the absence of granulomatous lesions of the respiratory tract.

The primary outcome studied was mortality. Secondary outcomes included the occurrence of treatment and disease related complications, time to first relapse, and number of relapses. All data are presented as medians and ranges.

We reviewed all articles listed on Pubmed describing the outcomes of paediatric vasculitis. This included both prospective and retrospective studies, observational data, case series, and case reports. Search terms included 'paediatric, vasculitis, Wegener's granulomatosis, microscopic polyangiitis, ANCA, churg strauss, and autoimmune'.

## Results

### Demographics

There have been a total of 48 paediatric patients diagnosed with ANCA vasculitis between the years of 1996 and 2010 at the Great Ormond Street Hospital. 5 of these patients were under adult follow up at the Hammersmith Hospital, London, with more than 10 years from initial diagnosis (Table 1). The University Hospital of Birmingham has a total of 220 patients with vasculitis under active follow up. 7 of these patients presented with vasculitis in childhood, 3 of whom had ANCA associated vasculitis and were under adult follow up with at least 10 years from initial diagnosis.

**Table 1:** Patient Demographics

Patient	Gender	Ethnicity	Diagnosis	Age at presentation	Follow up	Time to 1^st ^relapse(from diagnosis)	Total relapses
1	M	Caucasian	WG	11 years	25 years	56 months	4

2	F	Caucasian	MPA	14 years	30 years	2 months	4

3	F	Caucasian	WG	9 years	11 years	91 months	3

4	F	Afro-Caribbean	WG	9 months	27 years	186 months	6

5	F	Caucasian	WG	9 years	23 years	11 months	8

6	F	Afro-Caribbean	WG	12 years	14 years	Never reached full remission	2

7	M	Caucasian	WG	14 years	11 years	16 months	6

8	F	Caucasian	WG	15 years	12 years	24 months	1

Of the 8 patients, 2 were male and 6 were female. 6 patients were Caucasian, and 2 were African. Wegener's granulomatosis was the commonest diagnosis, present in 7 patients, while only 1 had MPA. The median age at presentation was 11.5 years (range 9 months to 15 years). Follow up time ranged form 11 to 30 years, with a median and mean of 19 years. Four patients were cANCA positive (all 4 with WG), 2 were pANCA positive (one with WG and one with MPA), and 2 were ANCA negative (both with WG).

### Clinical features

Arthralgia was the commonest clinical feature on initial presentation, present in 6 patients (Table 2). In addition, 5 patients presented with pulmonary disease with features of either intra- alveolar haemorrhage or cavitating pulmonary nodules.

**Table 2:** Clinical features on presentation

	1	2	3	4	5	6	7	8
Constitutional			+		+	+		

Fever				+	+			

Arthralgia/arthritis	+	+	+		+	+	+	

Renal impairment	+	+					+	+

Haematuria	+	+	+				+	+

Proteinuria							+	+

Skin lesions	+	+	+			+	+	

Neurological involvement						+		

Lung involvement	+	+					+	+

Haemoptysis						+	+	

Bloody stools						+		

Sinus involvement			+			+		

Eye involvement					+	+	+	

Epistaxis				+			+	

5 patients experienced renal disease with microscopic haematuria and 4 had acute renal failure on presentation. 1 patient was dialysis dependent (Patient 7), while renal function in the remaining patients was within the normal range. Four patients had ear nose and throat (ENT) symptoms of sinusitis, epistaxis, hearing loss or nasal disease at presentation. All of these patients had a clinical diagnosis of WG, while 1 of the patients also had tracheal stenosis and poor vocal cord movement (Patient 5).

Inflammatory eye symptoms were present in only 3 patients at presentation (with WG), manifesting as iritis and scleritis. Gastrointestinal symptoms were uncommon at presentation, being apparent in 1 patient with WG. Interestingly, 1 patient was classified as having juvenile idiopathic arthritis at presentation but was subsequently reclassified as having WG following the development of tracheal stenosis and a positive ANCA titre during disease flares (Patient 5). The diagnosis was confirmed by histology in four patients who had a renal biopsy that demonstrated pauci- immune focal necrotising glomerulonephritis.

In one patient, the initial presentation was consistent with polyarteritis nodosa(based on a positive angiogram, rash, and iritis) but she subsequently developed an MPA disease pattern, with pauci- immune glomerulonephritis and MPO-ANCA.

### Immunosuppression

All but one patient received cyclophosphamide during the course of their illness (Table 3). In general, a course of pulsed cyclophosphamide consisted of fortnightly doses of IV cyclophospahmide for a total of 3 doses followed by three weekly treatment for another 2-4 doses. A course oral cyclophosphamide (1-2 mg/kg/day) was for 3- 6 months but also depended on disease activity.

**Table 3:** Treatment summary

Patient	IV CP	Oral CP	IV MP	OSt	AZA/ MMF	Cy	Biologic Agents	PEX
1	+	-	-	+	+	-	-	-

2	+	+	-	+	+	+	-	-

3	+	-	+	+	+	-	Rituximab (x1)	9 cycles

4	-	-	-	+	+	+	-	-

5	-	+++	-	+	+	+	-	-

6	+	+	+	+	+	+	Rituximab (x2) Infliximab (x1) Campath (x1)	-

7	+	+	+	+	+	+	Rituximab (x4)	-

8	-	++	+	+	+	-	-	-

CP: Cyclophosphamide; MP: Methylprednisolone; OSt: Oral steroids; Cy: Cyclosporin; PEX: Plasma exchange

NB: '+' represents a single course of cyclophosphamide in the Oral Cyclophosphamide column

The one patient (with WG) who did not receive cyclophosphamide achieved remission with oral prednisolone alone. Of those treated with cyclophosphamide, 5 received more than one course. All patients received oral steroids, and 4 received additional pulsed intravenous methylprednisolone as a part of their induction therapy, which consisted of 500 mg methylprednisolone IV daily for 3 days followed be a tapering dose of oral steroids. Oral steroid exposure was difficult to ascertain due to the titration of doses during a treatment course. Steroid treatment consisted of a reducing dose of oral prednisolone starting at a dose of 1 mg/kg (up to 60 mg). Doses were reduced for the first 6 months down to 10 mg/day. This was tapered over the next 6- 12 months, depending on disease activity.

Biological agents used to achieve remission included rituximab only (in 2 patients with WG), and alemtuzumab, infliximab, and rituximab (all in 1 patient with grumbling WG). All biologic agents were given during adult follow- up, and none during paediatric care. All patients were maintained with either azathioprine or mycophenolate mofetil (MMF).

### Outcome

All but 1 patient achieved complete remission (Patient 6), 6 of who remained on long- term maintenance treatment. All patients experienced disease relapse, with a median of 4 episodes and range from 1 to 8 episodes (Table 1). The relatively frequent number of relapses may be explained by the fact that we included even minor relapses. However, it may also be explained by the possibility that the described severity of illness in our cohort is due to referral bias, which may concentrate more severe cases.

The median time to first relapse was 24 months (range 2 to 186 months). At a median follow up of 18.5 years (range 11-30 years) 1 patient had died (Patient 1), 25 years from initial presentation and following four relapses, from sudden death ascribed to respiratory complications of his WG.

Long- term complications of disease and treatment were significant (Table 4). Steroid induced side effects were a particular problem in these patients, including Cushing's syndrome (n = 2), osteoporosis (n = 2), and depression (n = 1). 1 patient underwent a hip replacement for avascular necrosis of the femoral head (Patient 5). Infections secondary to immunosuppression were clinically apparent in 7 of the 8 patients. 2 patients contracted shingles and 2 had candidiasis (oral, vaginal, and oesophageal). Respiratory tract, urinary tract, and skin infections were common. None of these episodes, however, were severe enough to warrant hospital admission and were treated successfully with a course of antibiotics as outpatients. Infertility was reported in 4 of the patients (3 females were amenorrhoeic and one male was infertile). 1 patient developed breast cancer at the age of 30 years (Patient 5).

**Table 4:** Disease and treatment related co- morbidities in patient cohort

Patient	Infertility	Infections	Musculo-skeletal and Skin	eGFR (ml/min)	Other
1	+	Suppurative skin infections Shingles	-	> 90	Cushing's syndrome Steroid- induced depression Chronic diarrhoea

2	+	Glandular fever Urinary tract infections Chest infection Otitis media Gastroenteritis	Alopecia	45	Nasal septal defect

3	-	Shingles Chest infections	-	> 90	-

4	+	Oral and vaginal thrush Fungal skin infection Urinary tract infection	-	72	Hearing impairment

5	+	Shingles Urinary tract infections Paronychia	Osteopoerosis AVN hip Cyclosporin- induced hirsuitism	> 90	Cushing's syndrome Hearing impairment Subglottic stenosis Breast Ca

6	-	Oeosphageal Candidiasis Chest infections	-	60	Pnemothorax post-emergency bronchial stenting Nasal septal defect Bronchial stenosis Bronchiectasis Hearing impairment

7	-	Recurrent chest and urinary tract infections	-	ESRD	-

8	-	-	Osteopoerosis	76	-

Long- term ENT complications were common in the WG patients, with 50% suffering from hearing impairment and nasal septal or upper airway deformities. Other significant treatment- associated complications included a pneumothorax following emergency bronchial stenting for severe sub-glottic stenosis.

Kidney function was generally well preserved, with follow up eGFR (Modified diet in renal disease; MDRD) between 45 and > 90 ml/min, with a median of 76 ml/min (excluding patient 7). Patient 7, who presented dialysis- dependent, remained so, until he received a renal transplant two and a half years after his initial diagnosis. The renal transplant was subsequently lost 8 months later due to poor adherence to therapy, and the patient was then re-established on haemodialysis.

Only 1 patient developed non-glomerular haematuria, that was secondary to endometriosis of the bladder wall. No other pre-malignant conditions were found and only one overt malignancy has been diagnosed in these 8 patients.

In summary, the overall disease and/or treatment related morbidity was high. Of the 8 patients, 1 patient died. All patients had relapsing disease; 7 suffered from infections, 4 were infertile, 2 had skeletal complications (osteoporosis or AVN of the femoral head); and 1 had psychiatric complications (depression), and 1 developed malignancy.

## Discussion

We describe the clinical features and long-term outcomes of 8 children diagnosed with ANCA vasculitis and followed up into adulthood for a median of 18.5 years (mean 19 years; range 11 to 30 years). In addition to studying our series of patients we reviewed a total of 7 reports that described the clinical course of 86 patients with paediatric onset of WG [3,6,9,10], MPA [11,12] and ANCA- associated vasculitis and necrotising glomerulonephritis (GN) [13] (Table 5). Particular emphasis was placed on treatment and complication rates. The follow up ranged from 4 months to 11 years. As the aim of our study was to describe the long- term follow up of paediatric patients with vasculitis, patients were only included in our series if they had more than 10 years of follow up.

**Table 5:** Summary of reported case series of paediatric patients with ANCA vasculitis

**MPA**	**n**	**M:F**	**Age**	**CKD**	**ESRD**	**Relapse rate**	**Rx**	**Deaths**
Bakkalogou [12]	10	4:6	Median: 12 years (8- 17)	4 (40%)	4 (40%)	5 episodes	Steroids Oral CP	0 (0%)

Peco- Antic [11]	7	1:6	Mean: 12 years (7- 15)	1 (14%)	2 (29%)	Not stated	Oral steroids Oral CP	0 (0%)

**Total MPA**	**17**	**5:12**	**7- 17 years**	**5** **(29%)**	**6** **(35%)**	**Not stated**	**-**	**0** **(0%)**

WG	n	M:F	Age	CKD	ESRD	Relapse rate	Rx	Deaths

Rottem [3]	23	7:16	Mean: 15.4 years (9- 19)	8 (35%)	2 (9%)	53% of all children and 38% of those with f/u > 5years had > 1 relapse	Oral pred and oral CP	2 (9%)

Stegmayr [6]	10	5:5	11- 30 years	6 (60%)	1 (10%)	Median: 28 months in 80%	9/10 patients received CP and steroids	0 (0%)

Belostotsky [9]	17	4:13	Mean: 6 years Median: 6.3 years (2 weeks - 14 years)	3 (18%)	0 (0%)	Not stated	All but one patient received CP + Pred. Pred in all patients	2 (12%)

Wong [10]	12	4:8	2 months to 14 years	Not stated	Not stated	Not stated	Not stated	Not stated

**Total WG**	**62**	**20:42**	**2 weeks to 30 years**	**17** **(27%)**	**3** **(5%)**	**Not stated**	**-**	**-**

AAV + Necrotising GN	n	M:F	Age	CKD	ESRD	Relapse rate	Rx	Deaths

Valentini [13]	7	2:5	13 years (+/- 0.9)	2 (29%)	1 (14%)	Not stated	IV MP + IV CP in all patients	0 (0%)

**Grand total**	**86**	**27: 59**	**2 weeks- 30years**	**24** **(28%)**	**11** **(12%)**	**-**	**-**	**-**

In the 86 reported patients, the age of disease onset ranged between 2 weeks and 16 years. However, 2 of the reports also included follow up of patients who presented as young adults [3,6]. We limited the upper age for inclusion in our cohort to 16 years. Only 2 out of our 8 patients (25%) were male. Similarly, there was a female predominance among the WG and MPA patients in the literature, with 68% and 70% of all patients being female respectively.

The long- term disease and treatment- related morbidity in WG remains a significant concern. Rottem et al reported a lower rate of treatment- related permanent morbidity in cyclophosphamide- treated paediatric WG patients compared to adult patients (22% vs 45%) [3]. Over a median period of 8.3 years, 48% of paediatric WG patients developed nasal deformities, 17% had hearing loss, 35% had subglottic stenosis, 35% had chronic kidney disease (9% with end stage renal disease (ESRD)), 48% had chronic sinus disease, 14% had pulmonary disease, and 9% were blind. Paediatric patients were 5 times more likely to have subglottic stenosis and twice as likely to have nasal deformities, favouring the use of more potent immunosuppression.

However, these patients also suffered from significant treatment related complications. Cystitis was present in 50% and infertility in 28% of patients receiving daily cyclophosphamide. 43% of patients had an average of 21 serious infections in 200 patient years. The relative risk of infection as a result of steroid treatment was 2, but rose to 7 with cyclophosphamide and 12 with both steroids and cyclophosphamide. There were no malignancies reported in this series [3]. Furthermore, Stegmayr reported regular relapses on stopping cyclophosphamide [6].

There are two studies describing childhood MPA [11,12]. Renal involvement appeared to be a predominant feature of MPA. Rapidly progressive glomerulonephritis occurred in 6 out of 10 patients in the series by Bakkaloglu [12]. Renal disease with biopsy- proven pauci- immune GN was present in all patients in a series by Peco- Antic [11]. The proportion of patients reaching ESRD was 40% and 29% respectively, with no deaths. Cyclophosphamide and steroids were used as induction therapy in both series. Neither series report long- term treatment related complications.

It is of interest that diagnostic categories may change with time. One of our cohort was initially diagnosed with polyarteritis nodosa (positive angiogram, negative ANCA) but subsequently their disease evolved into MPA, with evidence of pauci-immune glomerulonephritis and MPO-ANCA positivity.

The morbidity associated with current treatment is significant. Apart from one of our patients with WG who achieved remission with prednisolone, all of our patients received cyclophosphamide, with 5 patients receiving more than one course. We found a significant rate of infertility among our patients, with 4 of 8 (50%) patients affected. A slightly smaller number of patients (30%) in a review by Rottem et al suffered from infertility after cyclophosphamide treatment [3]. This is an obvious concern in the administration of cyclophosphamide in young patients. The co- morbidity associated with steroids is also significant. Rottem found a 2- fold increase in risk of infection with steroid treatment. Almost all of our patients had a steroid- related complication, particularly with long- term therapy.

The use of biologic agents is therefore a therapeutic strategy that needs exploring. Approximately half of our patients received biologic agents to control disease activity. 2 out of these 3 patients have suffered from significant infections subsequently. In a recent study by Eleftheriou et al, 25 paediatric patients with primary systemic vasculitis were treated with biologic agents including infliximab, rituximab, etanercept, and adalimumab. They found a significant improvement in BVAS score from 8.5 to 4 over a 32-month period. This was associated with a significant reduction in oral steroid requirement from 1.0 to 0.25 mg/kg/day. Notably, the overall rate of infection was 24%, but was more severe in those receiving infliximab [14].

In our review, we have included several papers from different centres with different treatment protocols, summarised in Table 5. However, it must be emphasised that our summary of the literature includes papers published over several years, with heterogeneity between patients and treatment protocols. This limitation must be considered when interpreting the quoted overall morbidity and mortality rates.

## Conclusions

We report significant late morbidity in 8 patients who presented with ANCA vasculitis in childhood. High relapse rates and treatment- associated complications including infection, steroid- related complications and infertility are of particular concern. Therefore, finding the most efficacious therapeutic regimen which is associated with the least side effects is especially important in paediatric vasculitis populations as the patients are likely to live long enough to develop late-therapy associated complications. In addition, although not yet observed in our series, late cardiovascular morbidity remains a major longer-term potential concern for adult survivors of paediatric vasculitis, an area of ongoing active research. Thus our results and those of others suggest that paediatric survivors of systemic vasculitis require careful long term follow up into adulthood with respect to ongoing significant disease activity and treatment related morbidity. Prospective cohort studies including paediatric patients are crucial to help us determine the best approach to managing this complex group of patients.

## Abbreviations

AAV: ANCA associated vascuitis; ANCA: Anti- neutrophil cytoplasmic antibody; BVAS: Birmingham vasculitis activity score; eGFR: Estimated glomerular filtration rate; ENT: Ear, nose and throat; ESRD: End stage renal disease; EULAR: European league against rheumatism; GN: Glomerulonephritis; HSP: Henoch Schonlein purpura; KD: Kawasaki disease; MMF: Mycophenolate mofetil; MPA: Microscopic polyangiitis; PAN: Polyarteritis nodosa; RPGN: Rapidly progressive glomerulonephritis; WG: Wegener's granulomatosis

## Competing interests

The authors declare that they have no competing interests.

## Authors' contributions

NA was involved in data gathering, writing the manuscript, performing the literature review, data analysis, and creating the tables. SJ was involved in data gathering. SS was involved in data gathering. LH is a senior author/editor and was involved in writing the manuscript. CDP is a senior author/editor and was involved in writing the manuscript. PB a senior author/editor, was involved in writing the manuscript, performing the literature review, and data analysis. ADS is a senior author/editor and was involved in writing the manuscrip, design of study, and data analysis. All authors read and approved the final manuscript.
